# Supplementary material for: Government Policy for the Procurement of Food from Local Family Farming in Brazilian Public Institutions
Source: Foods. 2021 Jul 10;10(7):1604. doi: 10.3390/foods10071604 (PMC8305705; doi:10.3390/foods10071604)
Supplement: Supplementary file 1 [file foods-10-01604-s001.zip › foods-1282912-SI.pdf]

Compra de alimentos em Restaurante institucional

Instituição compradora

*Obrigatório

1. 1. Em que tipo de instituição realiza suas atividades? *

Marcar apenas uma oval.

- Escolas *Pular para a pergunta 3*
- Universidade *Pular para a pergunta 3*
- Hospitais *Pular para a pergunta 3*
- Restaurantes populares *Pular para a pergunta 3*
- Forças armadas *Pular para a pergunta 3*
- Presídios *Pular para a pergunta 3*
- Outras *Pular para a pergunta 2*

2. Especifique em que tipo de instituição realiza suas atividades *

3. 2. Qual é o nome da instituição (escola, universidade, hospital, entre outros) em que realiza suas atividades?

4. 3. Qual a área de abrangência da instituição (escola, universidade, hospital, entre outros) em que realiza suas atividades? *

Marcar apenas uma oval.

- Municipal *Pular para a pergunta 6*
- Regional *Pular para a pergunta 6*
- Estadual *Pular para a pergunta 6*
- Nacional *Pular para a pergunta 6*
- Outros *Pular para a pergunta 5*

5. Especifique a área de abrangência da instituição *

6. 4. A instituição (escola, universidade, hospital, entre outros) é pública ou privada? *

Marcar apenas uma oval.

- Pública
- Privada
- Não sabe informar

7. 5. Estado *

8. *Marcar apenas uma oval.*

- Acre
- Alagoas
- Amapá
- Amazonas
- Bahia
- Ceará
- Distrito Federal
- Espírito Santo
- Goiás
- Maranhão
- Mato Grosso
- Mato Grosso do Sul
- Minas Gerais
- Pará
- Paraíba
- Paraná
- Pernambuco
- Piauí
- Rio de Janeiro
- Rio Grande do Norte
- Rio Grande do Sul
- Rondônia
- Roraima
- Santa Catarina
- São Paulo
- Sergipe
- Tocantins

8. 6. Nome da cidade *

9. 7. Qual é o cargo que você exerce na instituição? *

10. 8. No cargo que você exerce na instituição, participa de alguma das seguintes atividades? *

Marcar apenas uma oval por linha.

	Sim	Não	Não sabe informar
Elaboração da lista de compras	⬭	⬭	⬭
Planejamento do cardápio	⬭	⬭	⬭
Seleção de fornecedores	⬭	⬭	⬭
Compra de alimentos	⬭	⬭	⬭

CARACTERIZAÇÃO DO SERVIÇO DE ALIMENTAÇÃO DA INSTITUIÇÃO

11. 9. Qual a forma de gerenciamento do serviço de alimentação da instituição? *

Marcar apenas uma oval.

- ⬭ Terceirizada *Pular para a pergunta 13*
- ⬭ Autogestão *Pular para a pergunta 13*
- ⬭ Gestão mista *Pular para a pergunta 13*
- ⬭ Outra *Pular para a pergunta 12*

12. Especifique qual é a forma de gerenciamento do serviço de alimentação *

13. 10. A instituição conta com cozinha própria? *

Marcar apenas uma oval.

- Sim
- Não *Pular para a pergunta 14*

14. 11. Qual o número de pessoas que são atendidas diariamente pela instituição e que poderiam utilizar o serviço de alimentação? *

____

15. 12. A instituição oferece almoço? *

Marcar apenas uma oval.

- Sim *Pular para a pergunta 16*
- Não
- Não sabe informar

16. 13. Qual o custo aproximado para a instituição do almoço por pessoa por dia? *

____

17. 14. Qual o número almoços servidos diariamente pela instituição? *

____

18. 15. A instituição desenvolve ações para promoção da alimentação saudável/educação alimentar e nutricional (EAN)? *

Marcar apenas uma oval.

- Sim *Pular para a pergunta 19*
- Não *Pular para a pergunta 20*
- Não sabe informar *Pular para a pergunta 20*

19. 16. Quais ações para promoção da alimentação saudável/educação alimentar e nutricional são desenvolvidas? *

20. 17. A instituição desenvolve ações para promoção da alimentação sustentável? *

Marcar apenas uma oval.

- Sim *Pular para a pergunta 21*
- Não *Pular para a pergunta 22*
- Não sabe informar *Pular para a pergunta 22*

21. 18. Quais ações para promoção da alimentação sustentável são desenvolvidas? *

COMPRA DE ALIMENTOS

22. 19. Para abastecer o serviço de alimentação, são comprados alimentos de agricultores(as) familiares do município ou da região? *

Marcar apenas uma oval.

- Sim *Pular para a pergunta 23*
- Não
- Não sabe informar

23. 20. Em que ano a instituição iniciou a compra de alimentos da agricultura familiar? *

____

Pular para a pergunta 25

24. 21. A instituição teria interesse em comprar alimentos da agricultura familiar do município ou da região para abastecer o serviço de alimentação? *

Marcar apenas uma oval.

- ( ) Sim
- ( ) Não

Pular para a pergunta 39

22. Quais alimentos são comprados da agricultura familiar?

25. 22.1 Vegetais (legumes e/ou hortaliças) *

Marcar apenas uma oval.

- ( ) Sim *Pular para a pergunta 26*
- ( ) Não *Pular para a pergunta 27*
- ( ) Não sabe informar *Pular para a pergunta 27*

26. Quais vegetais (legumes e/ou hortaliças) são comprados? *

____
____
____
____
____

22. Quais alimentos são comprados da agricultura familiar?

27. 22.2 Frutas *

Marcar apenas uma oval.

- Sim *Pular para a pergunta 28*
- Não *Pular para a pergunta 29*
- Não sabe informar *Pular para a pergunta 29*

28. Quais frutas são comprados? *

____
____
____
____
____

22. Quais alimentos são comprados da agricultura familiar?

29. 22.3 Leguminosas (ex. Feijão) *

Marcar apenas uma oval.

- Sim *Pular para a pergunta 30*
- Não *Pular para a pergunta 31*
- Não sabe informar *Pular para a pergunta 31*

30. Quais leguminosas são comprados? *

____
____
____
____
____

22. Quais alimentos são comprados da agricultura familiar?

31. 22.4 Cereais *

Marcar apenas uma oval.

- Sim *Pular para a pergunta 32*
- Não *Pular para a pergunta 33*
- Não sabe informar *Pular para a pergunta 33*

32. Quais cereais são comprados? *

22. Quais alimentos são comprados da agricultura familiar?

33. 22.5 Carnes e ovos *

Marcar apenas uma oval.

- Sim *Pular para a pergunta 34*
- Não *Pular para a pergunta 35*
- Não sabe informar *Pular para a pergunta 35*

34. Quais carnes e ovos são comprados? *

22. Quais alimentos são comprados da agricultura familiar?

35. 22.6 Leite e derivados *

Marcar apenas uma oval.

- Sim *Pular para a pergunta 36*
- Não *Pular para a pergunta 37*
- Não sabe informar *Pular para a pergunta 37*

36. Quais leites e derivados são comprados? *

22. Quais alimentos são comprados da agricultura familiar?

37. 22.7 Alimentos processados (polpa de fruta, sucos, bolacha, geleia, pão, bolo, etc.). *

Marcar apenas uma oval.

- Sim *Pular para a pergunta 38*
- Não *Pular para a pergunta 39*
- Não sabe informar *Pular para a pergunta 39*

38. Quais alimentos processados são comprados? *

BENEFÍCIOS

39. 23. Em sua opinião, a compra de alimentos da agricultura familiar por instituições públicas pode gerar algum benefício? *

Marcar apenas uma oval.

- Sim
- Não
- Não sabe informar

24. Em sua opinião, quais desses são benefícios de comprar alimentos da agricultura familiar para o abastecimento dos restaurantes institucionais?

40. 24 a. Estimula a economia local *

Marcar apenas uma oval.

- Sim
- Não
- Não sabe informar

41. 24 b. Aumenta a quantidade de alimentos "in natura" produzidos na região *

Marcar apenas uma oval.

- Sim
- Não
- Não sabe informar

42. 24 c. Aumenta a variedade de alimentos "in natura" produzidos na região *

Marcar apenas uma oval.

- Sim
- Não
- Não sabe informar

43. 24 d. Aumenta a quantidade e a variedade de alimentos processados produzidos na região (polpa de fruta, sucos, bolacha, geleia, pão, bolo, etc.). *

Marcar apenas uma oval.

- Sim
- Não
- Não sabe informar

44. 24 e. Aumenta a oferta de alimentos frescos na instituição *

Marcar apenas uma oval.

- Sim
- Não
- Não sabe informar

45. 24 f. Aumenta a oferta de vegetais e de frutas no cardápio da instituição *

Marcar apenas uma oval.

- Sim
- Não
- Não sabe informar

46. 24 g. Contribui no resgate de tradições alimentares da região *

Marcar apenas uma oval.

- Sim
- Não
- Não sabe informar

47. 24 h. Melhora a qualidade da alimentação oferecida pela instituição *

Marcar apenas uma oval.

- Sim
- Não
- Não sabe informar

48. 24 i. Contribui para a sustentabilidade do sistema alimentar *

Marcar apenas uma oval.

- Sim
- Não
- Não sabe informar

49. 24 j. Aumenta a renda do agricultor *

Marcar apenas uma oval.

- Sim
- Não
- Não sabe informar

50. 24 k. Garante mercado para a comercialização de alimentos produzidos pelos agricultores familiares da região *

Marcar apenas uma oval.

- Sim
- Não
- Não sabe informar

51. 24 l. Outros benefícios, especifique.

DIFICULDADES

52. 25. Em sua opinião, existem dificuldades para realizar a compra/venda de alimentos da agricultura familiar para abastecer o serviço de alimentação de instituições (creches, escolas, restaurantes universitários, hospitais públicos)? *

Marcar apenas uma oval.

- Sim
- Não
- Não sabe informar

26. Em sua opinião, quais dessas são dificuldades para a compra/venda de alimentos da agricultura familiar da região para abastecer os restaurantes institucionais

53. 26 a. A demanda de alimentos dos restaurantes institucionais é maior que a capacidade de produção dos agricultores familiares da região. *

Marcar apenas uma oval.

- Sim
- Não
- Não sabe informar

54. 26 b. A sazonalidade da produção local não permite satisfazer a demanda de alimentos requeridos pela instituição, porque são solicitados alimentos que não estão disponíveis durante todo o ano. *

Marcar apenas uma oval.

- Sim
- Não
- Não sabe informar

55. 26 c. A compra/venda institucional de alimentos é um processo muito burocrático e pode demorar muito tempo para ser efetivada, o que dificulta que os agricultores ajustem os tempos de produção agrícola com a demanda da instituição. *

Marcar apenas uma oval.

- Sim
- Não
- Não sabe informar

56. 26 d. Os alimentos vendidos pelos agricultores familiares da região têm um custo mais elevado que aqueles comercializados por distribuidores. *

Marcar apenas uma oval.

- Sim
- Não
- Não sabe informar

57. 26 e. Os alimentos da agricultura familiar não são bem aceitos/recebidos pelas entidades consumidoras. *

Marcar apenas uma oval.

- Sim
- Não
- Não sabe informar

58. 26 f. Os critérios estabelecidos pela vigilância sanitária dificultam que a agricultura familiar da região comercialize seus produtos. *

Marcar apenas uma oval.

- Sim
- Não
- Não sabe informar

59. 26 g. A instituição compradora (escolas, hospitais, etc) não dispõe da infraestrutura necessária para o armazenamento de alimentos frescos. *

Marcar apenas uma oval.

- Sim
- Não
- Não sabe informar

60. 26 h. A falta de apoio da gestão pública e/ou do departamento de compras *

Marcar apenas uma oval.

- Sim
- Não
- Não sabe informar

61. 26 i. Falta informação da instituição compradora sobre a possibilidade de comprar alimentos da agricultura familiar. *

Marcar apenas uma oval.

- Sim
- Não
- Não sabe informar

62. 26 j. Falta informação por parte dos agricultores e suas organizações sobre a possibilidade de vender alimentos da agricultura familiar para instituições públicas. *

Marcar apenas uma oval.

- Sim
- Não
- Não sabe informar

63. 26 k. A falta de assistência técnica para os agricultores da região dificulta a produção e comercialização de alimentos. *

Marcar apenas uma oval.

- Sim
- Não
- Não sabe informar

64. 26 l. Os valores pagos pelas instituições públicas aos produtos da agricultura familiar são muito baixos. *

Marcar apenas uma oval.

- Sim
- Não
- Não sabe informar

65. 26 m. Existem poucos agricultores/as familiares na região *

Marcar apenas uma oval.

- Sim
- Não
- Não sabe informar

66. 26 n. Existem poucas organizações da agricultura familiar na região que comercializem alimentos. *

Marcar apenas uma oval.

- Sim
- Não
- Não sabe informar

67. 26 o. As organizações de agricultores não dispõem da infraestrutura necessária para o processamento de alimentos (descascar, cortar, embalar, etc) demandados pela instituição *

Marcar apenas uma oval.

- Sim
- Não
- Não sabe informar

68. 26 p. A logística de entrega dos produtos é muito custosa para as organizações de agricultores familiares e não compensa a venda para as instituições *

Marcar apenas uma oval.

- Sim
- Não
- Não sabe informar

69. 26 m. Outras dificuldades, especifique

Este conteúdo não foi criado nem aprovado pelo Google.

Google Formulários
